# Clinical characteristics and outcome of patients with acute mesenteric ischemia: a retrospective cohort analysis

**DOI:** 10.1007/s00423-021-02423-2

**Published:** 2022-01-19

**Authors:** Verena Martini, Ann-Kathrin Lederer, Jodok Fink, Sophia Chikhladze, Stefan Utzolino, Stefan Fichtner-Feigl, Lampros Kousoulas

**Affiliations:** Center of Surgery, Department of General and Visceral Surgery, Medical Center, University of Freiburg, Faculty of Medicine, Hugstetter Straße 55, 79106 Freiburg, Germany

**Keywords:** Acute mesenteric ischemia; Surgical therapy, Lactate, Treatment, Outcome, Mortality, Visceral surgery

## Abstract

**Background:**

Acute mesenteric ischemia (AMI) is an uncommon, but life-threatening clinical entity due to late diagnosis resulting in irreversible ischemic bowel necrosis. The most common causes of AMI are the embolic occlusion and the acute thrombosis of the mesenteric circulation. Typical treatment is composed of an early revascularization of the mesenteric circulation followed by abdominal surgery for resection of nonviable intestine and restoration of the intestinal continuity, but the mortality rates remain high.

**Methods:**

A retrospective cohort analysis was conducted, aiming to evaluate clinical characteristics, performed surgical procedures and outcomes of patients with acute mesenteric ischemia who underwent emergency abdominal surgery at a high volume surgical center in Germany.

**Results:**

Overall, 53 patients were identified with the intraoperatively proven diagnosis of AMI. Overall hospital mortality was with 62% comparable to the literature. Nineteen patients presented with an intraoperatively verified complete and non-reversible intestinal infarction without any angiographic or surgical option for a revascularization of the mesenteric circulation or an option for intestinal resection. From the rest of the patients, 14 underwent intestinal resection of the ischemic area without restoration of intestinal continuity; the other 20 underwent resection with a primary anastomosis to restore intestinal continuity. The mortality rate of these patients with curative-intended surgery remained high (41% of patients died). Pre- and postoperative hyperlactatemia were associated with lower survival of these patients.

**Conclusion:**

AMI remains a life-threatening abdominal emergency. Therapeutic approaches are highly depended on acting surgeon’s decision, being affected by subjectively rated bowel viability and physical condition of the affected patient. Only selected patients with good bowel viability appear to be suitable for receiving primary anastomosis. The results clearly indicate the need for further research to develop therapeutic approaches for a better management of AMI and to improve outcome of affected patients.

## Background

Acute mesenteric ischemia is defined as a sudden interruption of the blood supply to a segment of the small intestine and is a rare cause of abdominal emergency characterized by severe abdominal pain and rapid disease progression, leading to intestinal necrosis, intestinal infarction, and patient death if untreated (1, 2). The mortality rate extends up to 70% due to late diagnosis and fatal development of intestinal necrosis and infarction (3). The most common pathogenic mechanism is the embolic occlusion of the superior mesenteric artery (SMA). Other causes can be arterial and venous thrombosis and non-occlusive causes (NOMI) (2, 4). NOMI originates from a vasoconstriction of the splanchnic arteries without an underlying structural stenosis (2). Elderly patients and patients with pre-existing cardiac diseases are at increased risk of developing acute mesenteric ischemia (AMI) (4, 5). Due to varying symptoms and lack of physical findings of peritonitis in the early phase of the ischemia, diagnosis is very challenging (6, 7). Computed tomography angiography can rapidly and accurately confirm the diagnosis of AMI and is the modern gold standard for diagnosis, complemented by serological parameters including leukocyte counts, pH, and lactate values which however lack the sensitivity and specificity to exclude acute mesenteric ischemia (1, 8). Acute mesenteric ischemia requires emergency surgery. Typical treatment consists of early revascularization of the mesenteric circulation followed by abdominal surgery for resection of the infarcted areas of the intestine (1, 9). Current surgical recommendations focus on damage control surgery and favor a second look operation for a definitive therapy (1). So far, the chosen surgical approach during first operation is based on patients’ presentation as well as on the individual experience and preference of the surgeon. A critical retrospective evaluation of treatment is needed to improve further therapeutic approaches. Therefore, the aim of this study was to evaluate clinical characteristics, risk factors, type of performed surgical procedures, and outcomes of patients with acute mesenteric ischemia who underwent emergency surgery at a high volume surgical center in Germany.

## Methods

From January 2010 to December 2017, all patients with acute mesenteric ischemia, who underwent abdominal surgery at the author’s institution, were retrospectively screened. The study was approved by the ethical committee of the Medical Faculty of the University of Freiburg and was registered in the German Clinical Trial Register.

The main inclusion criterion was the intraoperative verification of AMI. Patients with a secondary ischemia due to ileus or adhesions as well as patients with aortic dissection or isolated dissection of the SMA were excluded from this study.

All data was obtained from in-house medical records. The examined parameters included patients’ demographics, pre-existing diseases, type of performed operative procedures, overall survival, the time interval from hospital admission to CT and to surgery as well as the lactate value at hospital admission, and the first postoperatively measured lactate value. Intraoperative variables collected included the type and region of resection and the type of reconstruction of the intestinal continuity.

Blood samples for lactate measurement were taken from an arterial catheter and were processed immediately. Arterial serum lactate was analyzed at bedside via blood gas analysis. Lactate value of 1.7 mmol/l and above was defined as hyperlactatemia. Venous blood samples for measurement of leukocytes and procalcitonin (PCT) were also processed immediately. Measurement of concentration of leukocytes and PCT was performed by Central Laboratory of the University Medical Center of Freiburg, Germany. Leukocytosis was defined as a leukocyte count of more than 10 thousand/µl, and PCT above normal range was defined as > 0.05 ng/ml.

### Statistical analysis

Exploratory statistical analysis was conducted using IBM SPSS for Windows (version 27.0). Continuous data were expressed in the form of mean and standard deviation. Categorical data were expressed as number and percentage. Chi-square test as well as the Fisher’s exact test for small sample sizes was used for group comparison. Mann–Whitney *U* test was used for comparison of non-normal-distributed metrical parameters. Analysis of affecting factors on mortality rate was performed using the multiple logistic regression analysis. Repeated measures ANOVA was used to evaluate the impact of survival status on laboratory parameters. *P*-values less than 0.05 were considered significant.

## Results

Between January 2010 and December 2017, eighty patients were operated on with the primary radiologic diagnosis of acute mesenteric ischemia. Out of these 80 patients, 53 met the inclusion criteria and were retrospectively analyzed (Table 1). Reasons for exclusion were intraoperatively verified other diagnoses (no AMI) or secondary ischemia due to ileus, strangulated hernia or adhesions or a preoperatively diagnosed dissection of aorta or SMA. All of the included patients had diagnosis of suspected AMI preoperatively, which was intraoperatively verified. Suspected diagnosis was delivered by CT scan in 50 patients (94%) and by sonography in 3 patients (6%).

**Table 1:** Patients’ characteristic (*SD* standard deviation, *AMI* acute mesenteric ischemia, *NOMI* non-occlusive mesenteric ischemia)

Sex (*n* male/female, %)	34/19 (64%/36%)
Age (years, SD)	74 ± 11.4
**Common comorbidities** - Hypertension - Atrial fibrillation - Coronary heart disease - Peripheral vascular disease - Congestive heart failure - Diabetes - Renal insufficiency - Prior myocardial infarction	***n* (%)** 32 (60%) 26 (49%) 24 (45%) 20 (38%) 13 (25%) 16 (30%) 13 (25%) 10 (19%)
Interventional embolectomy before surgery (*n*, %)	7 (13%)
**Antibiotic treatment before intervention** Empiric therapeutic antibiotics, immediately after diagnosis Single shot prior to surgery	***n* (%)** 40 (71%) 56 (100%)
**Therapeutic anticoagulation preoperatively** Heparin i.v No anticoagulation prior to surgery	***n* (%)** 38 (72%) 14 (21%)
**Type of AMI** Arterial thrombosis Arterial embolism Venous thrombosis NOMI	***n* (%)** 23 (43%) 21 (40%) 2 (4%) 7 (13%)
**Bowel affected by AMI** Colon Small bowel Small bowel and colon	***n* (%)** 17 (32%) 22 (42%) 14 (26%)
**Type of operation** Exploratory laparotomy without resection/intervention Thrombectomy of superior mesenteric artery Subtotal gut resection Hemicolectomy right Colectomy Ileocecal resection Segmental ileal resection Segmental jejunal resection	***n* (%)** 19 (36%) 1 (2%) 2 (4%) 11 (21%) 6 (11%) 3 (6%) 6 (11%) 5 (9%)
**Type of anastomosis (*n* = 20)** Jejunotransversostomy Jejunojejunostomy Ileotransversostomy Ileoileostomy Jejunoileostomy Ileoascendostomy	***n* (%)** 1 (5%) 4 (20%) 6 (30%) 4 (20%) 4 (20%) 1 (5%)
**Technique of anastomosis (*n* = 20)** Side-to-side End-to-end Not specified	7 (35%) 11 (55%) 2 (10%)
**Type of ostomy (*n* = 13)** Terminal ileostomy Terminal jejunostomy Terminal transversostomy	10 (77%) 2 (15%) 1 (8%)
**Lactate level** - At admission (*n* = 44) - After operation (*n* = 34)	**mean ± SD (mmol/l)** 4.3 ± 4.0 3.7 ± 4.0
**Leukocytes** - At admission (*n* = 53) - After operation (*n* = 34)	**mean ± SD (thousands/µl)** 15.5 ± 7.9 13.8 ± 6.8
**Procalcitonin** - At admission (*n* = 23) - After operation (*n* = 16)	**mean ± SD (ng/ml)** 9.4 ± 8.5 15.0 ± 14.7
**Mortality (*n*, %)**	33 (62%)

Most of the patients had arterial thrombosis (*n* = 23, 43%), followed by arterial embolism (*n* = 21, 40%) and NOMI (*n* = 7, 13%). Venous thrombosis was only found in 2 patients (4%). More than half of the patients suffered from arterial hypertension, followed by other cardiologic diseases such as atrial fibrillation, coronary heart disease, or peripheral vascular disease (Table 1). Only 5 patients did not suffer from common comorbidities. Forty-six patients (87%) were referred from surrounding hospitals. The distance to the referring hospital was on average 29 km (0–82 km). The majority of patients (44%, *n* = 20) were transferred from a cardiology center. Twenty-five patients stayed longer than 24 h in the referring hospital, 10 of whom were admitted due to other elective indications. None of the patients had an aortography or coronary arteriography immediately (< 48 h) before the transfer to our hospital. The time between first signs of AMI and referring is not known, but patients who were initially admitted to another hospital receiving CT scan in the other hospital had a significantly longer time interval between CT scan and surgery than directly to our hospital admitted patients (492 vs. 167 min, *p* < 0.001).

The majority of patients received a therapeutic empirical antibiotic treatment (72%) (*n* = 38), mostly penicillin and a β-lactamase inhibitor immediately after diagnosis, but before the surgery.

Patients not being treated with antibiotics therapeutically were given a standard prophylactic shot of antibiotics immediately before surgery.

A total number of 12 patients (23%) were not treated with antibiotics preoperatively. Seven of these patients suffered from an infaust ischemia.

Regarding the therapeutic anticoagulation, 72% (*n* = 38) of the cohort were given heparin i.v. even before the surgical approach. In four cases, no data was available. In 21% (*n* = 11), no systemic intravenous anticoagulation was administered before the operation. In 43% of these cases, the patients received coagulation products prior to surgery due to derailed coagulation. Almost a third of the patients not receiving heparin i.v. had a preexisting oral anticoagulation due to other comorbidities.

Seven patients received an intervention prior to surgery; of those, three survived.

The overall in-hospital mortality was 62% (*n* = 33). Nineteen patients (36%) had an intraoperatively verified complete and irreversible bowel ischemia limiting the therapeutic approach to palliative care. Furthermore, 14 patients (41% of all patients with curative-intended surgery) died after surgery due to abdominal sepsis (*n* = 8), progression of ischemia (*n* = 3), or pneumonia (*n* = 3).

Multiple logistic regression did not reveal any factors affecting mortality of patients (Table 2).

**Table 2:** Multiple logistic regression of all patients (*n* = 53) (*AMI* acute mesenteric ischemia, *NOMI* non-occlusive mesenteric ischemia; Nagelkerke’s *R*^2^ = 0.531)

Parameter	*p*	Odds	95% confidence interval	
			Upper	Lower
Age (years)	0.244	1.082	0.948	1.234
Sex (male/female)	0.425	2.442	0.272	21.919
Comorbidity (yes/no)	0.598	0.206	0.001	72.921
Referred from another hospital (yes/no)	0.854	0.722	0.022	23.347
In-house CT scan (yes/no)	0.813	0.727	0.051	10.270
Anastomosis (yes/no)	0.116	0.155	0.015	1.587
**Type of AMI**				
Arterial thrombosis	0.734	2.532	0.012	540.039
Arterial embolism	0.809	0.452	0.001	278.492
NOMI	0.529	9.061	0.009	8689.024
Venous thrombosis	Reference	Reference	Reference	Reference
**Bowel affected by AMI**				
Colon	0.454	0.266	0.008	8.540
Small bowel	0.163	0.118	0.006	2.373
Colon and small bowel	Reference	Reference	Reference	Reference
**Preoperative laboratory values**				
Hyperlactatemia (yes/no)	0.166	5.174	0.505	52.987
Leukocytosis (yes/no)	0.615	1.780	0.188	16.870

### Surgery

All patients underwent open abdominal emergency surgery (types of operation are shown in Table 1). AMI affected the small bowel in 42% of patients (*n* = 22) and the colon in 32% of patients (*n* = 17). Combination of both was found in 26% of patients (*n* = 14). Overall, 19 patients had an intraoperatively verified complete and irreversible ischemia of the small bowel (*n* = 8) or of the small gut and colon (*n* = 11) leading to an abortion of exploratory laparotomy without bowel resection. Four of these patients experienced an endovascular intervention. Intraoperative viability was tested by clinical examination and pulsation as a subjective assessment.

Acting surgeons reported viability of bowel in 19 cases as “not restorable,” in 21 cases as “good,” and in 6 cases as “adequate.” In 7 cases, nothing was reported about seen viability. In one patient, intraoperative arteriography of superior mesenteric artery (SMA) and in one further patient, intraoperative Doppler sonography of SMA showed sufficient blood flow. Acting surgeons described pulsation of different mesenteric vessels (80% SMA) as “insufficient” in 19 patients and as “good” in 10 patients. In 23 patients, nothing was reported about pulsation of mesenteric vessels.

Out of 34 patients with curative-intended surgery, 13 patients received discontinuity resection (9 patients received end ileostomy, 2 patients received end jejunostomy, and 1 patient received end transverse colostomy, further 1 patient did not receive ostomy leading to a second look operation). Anastomosis was performed in 20 patients (types of anastomoses are shown in Table 1). Most of these patients had subjectively rated “good” bowel viability: Perfusion was described as “adequate” in only two cases; in one case, status of viability was not reported. An anastomosis was significantly more frequently performed in patients with small bowel resections (*n* = 13, 85% anastomoses) than in patients with colon resection (*n* = 20, 45% anastomoses; *p* = 0.026). Postoperative leakage of anastomosis occurred in 2 patients (10% of anastomoses). In 1 patient, resection of the bowel was not necessary due to complete restoration of circulation after intraoperative thrombectomy of an SMA thrombosis. Overall, 2 patients received successful intraoperative thrombectomy. Intraoperative embolectomy was performed in 3 patients, with only 2 successful results.

Patients with small bowel ischemia (*n* = 15, 44%) needed on average a resection of 52 cm (range 10–150 cm) of small intestine. In 3 patients, extensive resection of the small bowel was necessary leading to a remaining length of small bowel of 150, 180, and 200 cm.

### Impact of laboratory values on mortality rate

**Fig. 1:**
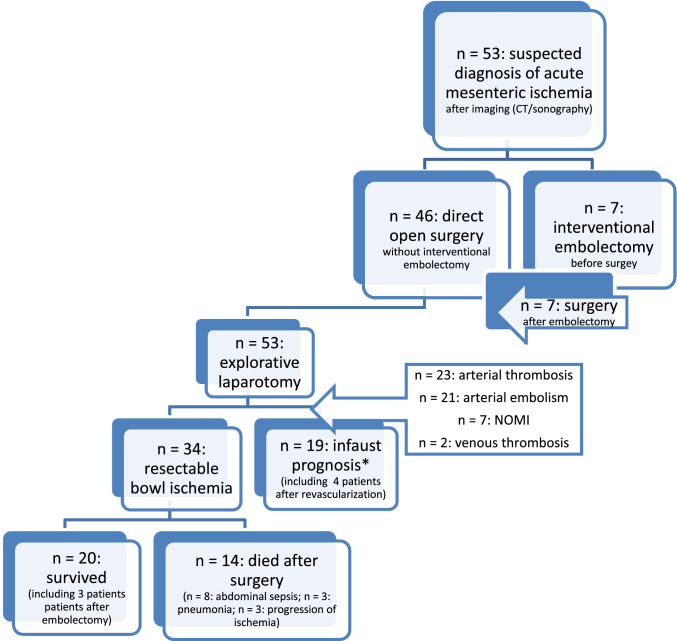
Flowchart of included patients (*intraoperatively verified complete and irreversible ischemia of the small bowel (*n* = 8) or of the small gut and colon (*n* = 11); NOMI, non-occlusive mesenteric ischemia)

Lactate value was measured in 44 patients at admission and in 34 patients after surgery (Table 3). Preoperative lactate concentration was significantly increased in patients, who died postoperatively (5.2 mmol/l vs. 2.7 mmol/l, *p* = 0.019). Thirty-three (75%) patients had hyperlactatemia (> 1.7 mmol/l) at admission; more than 70% of these patients (*n* = 24) died, whereas mortality rate in patients without preoperative hyperlactatemia was 35% (*p* = 0.037). The postoperative lactate concentration was significantly higher in patients, who died (5.8 vs. 2.2 mmol/l, *p* = 0.004). Repeated measures ANOVA confirmed the observation and revealed an impact of survival status on lactate level (*p* = 0.031, Fig. 1).

**Table 3:** Comparison of lactate level, concentration of leukocytes and procalcitonin pre- and postoperatively in survivors (*n* = 20) and deceased (*n* = 33) (*SD* standard deviation)

	Survived	Deceased	*p*
**Lactate level (mean ± SD [mmol/l])** - At admission - After operation	2.7 ± 2.3 2.2 ± 2.0	5.2 ± 4.6 5.8 ± 5.1	0.019* 0.004*
**Leukocytes (mean ± SD [thousands/µl])** - At admission - After operation	13.6 ± 8.5 12.1 ± 6.4	16.6 ± 7.4 16.2 ± 6.8	0.090 0.039*
**Procalcitonin (mean ± SD [ng/ml])** - At admission - After operation	7.8 ± 9.3 21.9 ± 17.9	10.4 ± 8.2 9.5 ± 9.3	0.250 0.210

P-values less than 0.05 were considered significant and marked bold and *

**Fig. 2:**
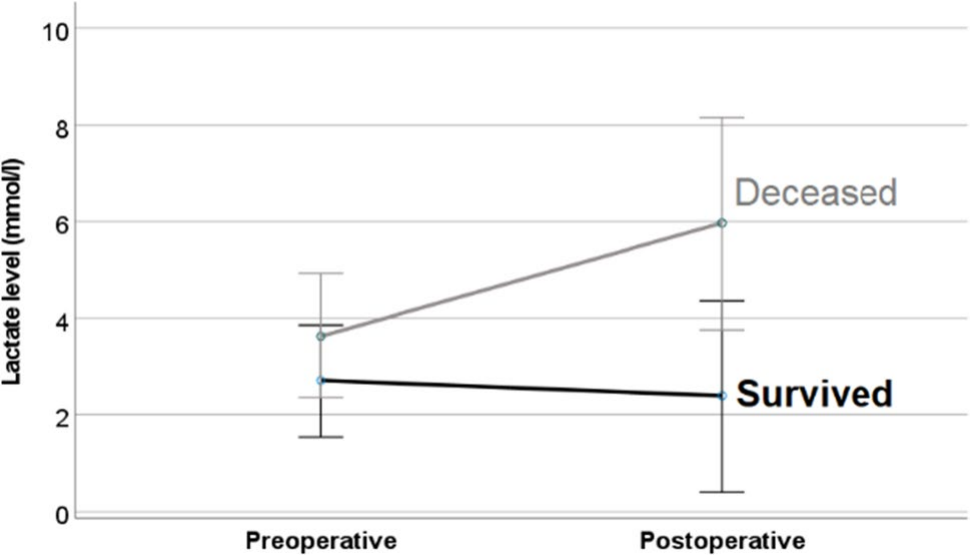
Comparison of course of lactate level of deceased and survivors. Repeated measures ANOVA revealed an impact of survival status on lactate level (*p* = 0.031)

Leukocytes were measured in all patients preoperatively and in all patients with curative-intended surgery (*n* = 34) postoperatively (Table 3). The concentration of leukocytes did not differ significantly between survivors and deceased preoperatively. Postoperatively, survivors showed a slightly lower concentration of leukocytes than diseased (12.1 vs. 16.2 thousands/µl, *p* = 0.039). Leukocytosis occurred in 40 of patients (76%) preoperatively and in 25 of patients (47%) postoperatively, but there was no difference of occurrence of leukocytosis between survivors and deceased. Repeated measures ANOVA did not reveal an impact of survival status on leukocyte count (Fig. 2).

Procalcitonin (PCT) was measured in 23 patients pre- and in 16 patients postoperatively (Table 3). The concentration of PCT did not differ significantly between survivors and deceased neither pre- nor postoperatively. All of the patients had values exceeding normal range (< 0.05 ng/ml) pre- and postoperatively. Repeated measures ANOVA did not reveal an impact of survival status on concentration of PCT.

## Discussion

Acute mesenteric ischemia is a rare but life-threatening clinical entity with vague symptoms, leading to a high mortality due to delayed diagnosis. Our study is in line with previous data in terms of an in-hospital mortality of 62% (4, 7, 10). Besides patients, who had an intraoperatively verified complete and not-restorable bowel ischemia implying certain death, the mortality of our patients with curative-intended surgery was still more than 40%, which might also be caused by the fact that AMI is a disease preferably affecting the elderly and those with pre-existing illnesses (4, 5). Most of our patients were transferred from a cardiologic center implying pre-existing cardiac illness as a risk factor for development of AMI. Interestingly, our patients were transferred without an interventional procedure shortly before development of AMI, but recent research suggests plausibly that vascular manipulation can precipitate AMI (11, 12).

AMI is a rapidly progressing disease, implacably resulting in irreversible bowel ischemia, bowel infarction, and patient death if not treated in a timely manner (2, 13). Therefore, it is widely stated that urgent imaging in case of suspected AMI is of paramount importance (8, 14). Unfortunately, as the time of patients’ symptom development and admission in most primary care centers was not documented, we cannot judge on this issue. Indeed, patients from primary care hospitals needed 7 h longer from diagnostics to surgery, but mortality rates of directly admitted and transferred patients are not robustly comparable as the low sample size of only 7 not-transferred patients leads to statistical non-valid results. Due to a varying symptomology in the beginning, diagnosis of AMI is challenging (6, 7, 11). The small bowl system and the colon can be equally affected (1). Lactate levels are used to support suspected diagnosis (15, 16). In our cohort, the majority had a hyperlactatemia at admission and serum lactate could be identified as prognostic marker for mortality. Higher lactate levels are known to be associated with poor prognosis (17, 18). Postoperative lactate levels of our resectable patients were higher in those who died during the postoperative course. We also found a tendency of higher levels of leukocytes in patients with poor prognosis, which is also in line with previously published data of other groups (7, 19, 20). PCT was elevated in all of our patients, reflecting the diagnosis of AMI and the deterioration of general condition.

Once the diagnosis of AMI is confirmed, the choice of therapy is an open abdominal surgery.

Revascularization is the primary goal and interventional radiology for early revascularization of the mesenteric circulation as well as open vascular surgery might accompany the visceral approach (1, 9, 21).

Immediate antibiotic treatment and anticoagulation are recommended to complement the surgical approach (11). Laparotomy is pivotal to evaluate the extent of visceral organ ischemia (10, 22). Suspected AMI regularly needs emergency operation, leaving the surgeon on duty in charge of the best approach for his patient. The assessment of small bowel and colon viability and the evaluation of patients’ condition appear to be crucial for choosing the right surgical approach (11). The results of our retrospective analysis indicate a distinctly surgeon-depended therapeutic approach for treatment of AMI, as intraoperative viability was mostly subjectively rated and approach was chosen by eminence. Recent recommendations, firstly published in 2016 and 2017, focus on damage control surgery and favor a second look operation for a definitive therapy in critical ill patients (1, 11). Performing of an anastomosis is only recommended in stable patients without any sign of shock or multiple organ failure (11). However, the recommendations are just based on a few studies indicating that AMI research is lacking of evidence. As mentioned above, assessment of bowel viability was mostly a subjective rating by acting surgeons, which is in line with the recent recommendation as assessment of bowel viability should be based on macroscopic bowel appearance such as color, moving, and bleeding (11). Decision-making can be supported by intraoperative techniques such as Doppler ultrasound or use of fluorescein (11, 23). In our cohort, it was interesting that only a minority of the patients met the radiologic inclusion criteria for a possible interventional approach. Furthermore, it is noteworthy that the verification of the intraoperative blood flow is not routinely stated. It is pivotal to improve the surgical awareness that the revascularization is the major goal.

The results of our study are limited due to incomplete documentation caused by the retrospective study character. The small sample size of our study shows the difficulty of AMI research as AMI remains a rare abdominal disease occurring just in a small patient cohort.

## Conclusion

AMI remains a life-threatening abdominal emergency with a high mortality rate. Therapeutic approaches are highly dependent on the performing surgeon’s decision, being affected by subjectively rated bowel viability and physical condition of the affected patients. Only the selected patients with good bowel viability appear to be suitable for receiving primary anastomosis. Nevertheless, the results clearly indicate the need for further research to develop therapeutic approaches for a better management of AMI and to improve outcome of the affected patients.

## Abbreviations

AMI: Acute mesenteric ischemia
NOMI: Non-occlusive mesenteric ischemia
PCT: Procalcitonin
SD: Standard deviation
SMA: Superior mesenteric artery
